# Synergistic Catalysis over MoS_2_/CuS in Ultrasound-Assisted Peroxymonosulfate System: Performance and Mechanism for Degradation of Multiple Organic Contaminants

**DOI:** 10.3390/molecules31183210

**Published:** 2026-09-11

**Authors:** Chu Dai, Jie Li, Chuanhui Wang, Hongyan Qi, Chen Tian

**Affiliations:** 1 College of Advanced Energy Materials, Hubei University of Education, Wuhan 430205, China; 2 Hubei Engineering Technology Research Center of Environmental Purification Materials, Institute of Materials Research and Engineering, Hubei University of Education, Wuhan 430205, China; 3 Yangtze River Fisheries Research Institute, Chinese Academy of Fishery Sciences, Wuhan 430223, China

**Keywords:** peroxymonosulfate, piezocatalysis, ofloxacin, MoS_2_/CuS, water remediation

## Abstract

Aquatic antibiotic pollution represented by ofloxacin (OFX) causes serious ecological hazards and endangers public health due to the high persistence and bioaccumulation of antibiotic residues. Conventional water treatment techniques are insufficient for OFX elimination, limited by low removal efficiency, high energy consumption, and poor operational stability. Herein, a novel MoS_2_/CuS heterojunction composite was fabricated via a hydrothermal method and applied to an ultrasound-driven piezocatalysis-coupled peroxymonosulfate (PMS) advanced oxidation system for OFX wastewater remediation. The introduction of CuS effectively remedies the inherent shortcomings of pristine MoS_2_, including insufficient active sites and rapid photogenerated carrier recombination. The constructed heterojunction induces a strong interfacial built-in electric field, which significantly accelerates the migration of piezoelectric charges. The synergistic photo-piezoelectric effect further promotes continuous PMS activation and facilitates the massive generation of reactive oxygen species (ROS). The influences of key operating parameters and common water inorganic anions on OFX degradation performance were systematically investigated. Radical trapping experiments confirmed the synergistic mechanism between piezocatalysis and PMS activation during the catalytic reaction. The optimized MoS_2_/CuS heterojunction exhibits remarkable OFX degradation efficiency and excellent cyclic stability. This work provides a feasible strategy for the rational design and fabrication of high-efficiency piezocatalysts and offers a promising technical route for the remediation of refractory antibiotic wastewater via piezocatalysis-coupled PMS advanced oxidation.

## 1. Introduction

Given the rapid advancement of the modern pharmaceutical industry, antibiotics have been extensively employed in medical treatment, livestock breeding and other fields owing to their broad antibacterial spectrum and remarkable therapeutic effects [1,2,3]. In particular, ofloxacin (OFX), as a typical fluoroquinolone, is frequently detected in natural waters. These compounds are poorly biodegradable and readily accumulate in aquatic environments. Consequently, they not only disrupt the balance of aquatic ecosystems but also may induce the emergence of antibiotic resistance genes in bacteria, posing potential risks to human health [4,5]. Conventional remediation methods, such as adsorption and biological treatment, are poorly equipped to eliminate such persistent pollutants [6,7]. Advanced oxidation processes (AOPs) offer an alternative; they generate highly reactive radicals that can efficiently mineralize organic molecules, which has sparked intense research interest [8,9,10]. Among them, persulfate-based AOPs are especially attractive due to their mild reaction conditions, strong oxidation capacity and wide application range [11,12].

To activate persulfate, the key step is to generate reactive oxygen species [13,14]. Conventional methods like thermal or photoactivation, however, are energy-intensive, demand harsh conditions, and often suffer from poor catalyst stability. These drawbacks seriously hinder their practical applications. Piezocatalysis offers a greener route: it uses mechanical stress (like ultrasound) to build an internal electric field in piezoelectric materials [15,16,17]. That field separates electrons and holes, which then react with persulfate to break down pollutants. This strategy is gaining attention as a promising alternative. For instance, Meng and co-workers reported a tribo-positive Fe@MoS_2_ piezoelectric catalyst prepared by a mechanochemical method, which showed notable piezoelectric catalytic performance [18]. No obvious performance decay was observed after 50 consecutive cycles, where the tetracycline degradation efficiency of Fe@MoS_2_ remained over 99%, revealing its excellent cycling durability.

As a typical two-dimensional layered piezoelectric material, MoS_2_ possesses favorable piezoelectric response and catalytic activity, rendering it a promising candidate for piezocatalysis–persulfate systems [19,20,21,22,23]. Nevertheless, pristine MoS_2_ suffers from low carrier separation efficiency and insufficient active sites, restricting its persulfate activation ability and pollutant removal performance for practical treatment [24,25]. Constructing heterojunction composites is an effective strategy to overcome the performance limitations of bare MoS_2_ and boost piezocatalytic activity. CuS is a narrow-bandgap semiconductor with excellent electrical conductivity, distinctive localized surface plasmon resonance effect and favorable photothermal conversion capacity [26,27,28,29,30,31]. In addition, the variable valence states of copper ions can participate in multi-electron reduction processes, which enables the fabrication of high-efficiency heterojunctions with MoS_2_. When CuS is integrated with MoS_2_ to form MoS_2_/CuS heterojunctions, the interfacial built-in electric field facilitates the separation and migration of piezoelectrically induced charge carriers. Meanwhile, the photothermal property of CuS can couple with the piezoelectric effect to realize photo-piezo synergistic catalysis, further enhancing persulfate activation and the generation of reactive oxygen species. The construction of this composite system provides a new avenue for designing high-performance piezocatalysts and offers an alternative strategy for remediating refractory antibiotic contamination in complex water matrices.

Accordingly, this work designed and synthesized MoS_2_/CuS composites, which were applied in an ultrasound-driven piezocatalysis-coupled persulfate system for the remediation of OFX-contaminated water. Monomeric MoS_2_ and MoS_2_/CuS heterojunction catalysts were fabricated via a hydrothermal method combined with calcination. The physicochemical properties of the materials were systematically characterized. To evaluate the catalytic system, we chose OFX as the target pollutant and examined the influence of key reaction parameters and inorganic anions on degradation efficiency. Subsequently, radical quenching tests were carried out to elucidate the synergy between piezocatalysis and persulfate activation, shedding light on the interfacial charge transfer and the overall cooperative effects. This study aims to provide theoretical guidance and experimental support for the design of high-efficiency and stable broad-spectrum responsive piezocatalysts and promote the practical application of piezocatalysis–persulfate technology in the remediation of complex aquatic environments.

## 2. Results and Discussion

### 2.1. Characterization of MoS_2_/CuS Catalyst

**Figure 1:**
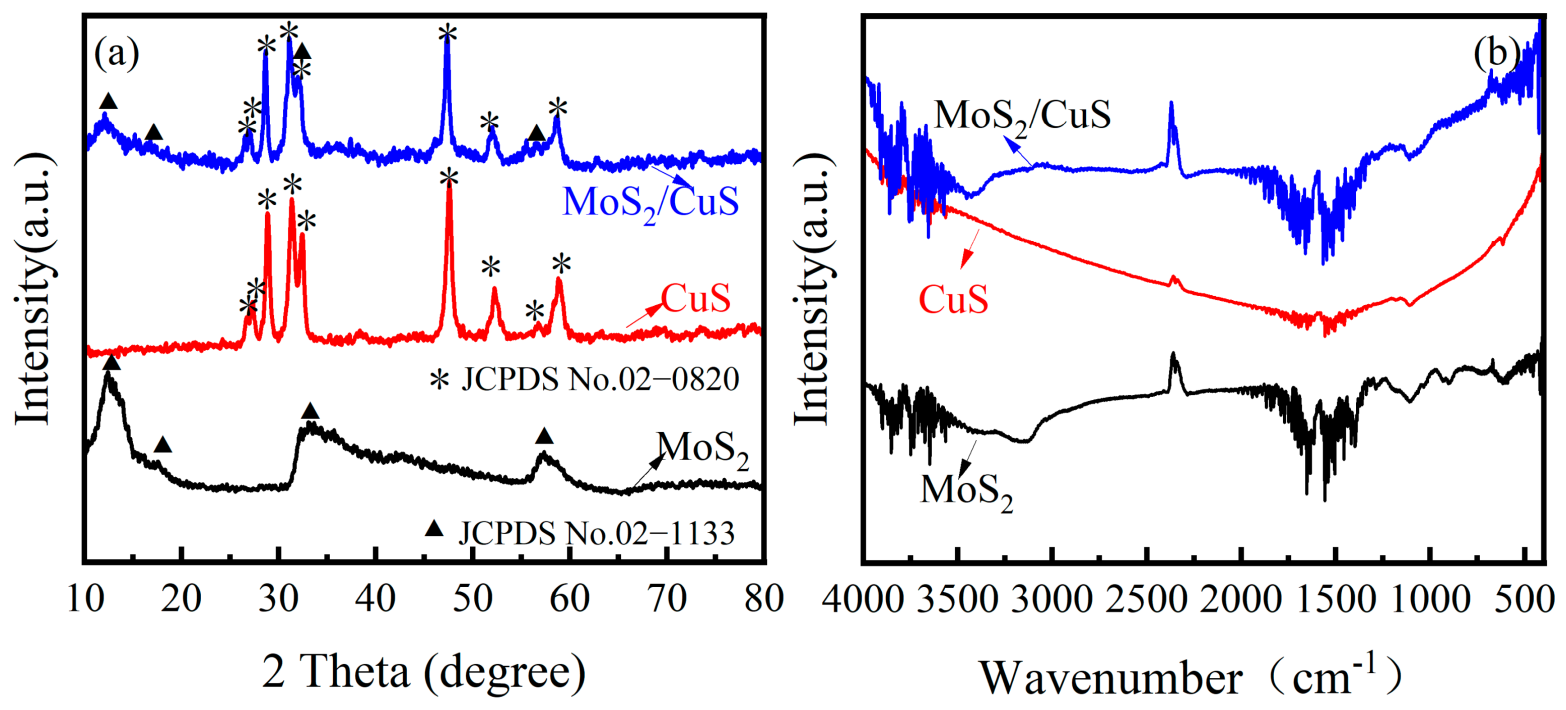
(**a**) XRD patterns and (**b**) FTIR spectra of various catalysts.

The crystal structure of the prepared materials was analyzed via XRD, and the corresponding patterns are displayed in Figure 1a. In the 2θ scanning range of 10–80°, pure CuS displayed well-defined characteristic diffraction peaks at 26.75°, 27.51°, 29.36°, 31.82°, 33.03°, 48.10°, 52.88°, and 59.18°, while pure MoS_2_ showed typical diffraction signals at 13.38°, 15.73°, 33.67°, and 58.36°. These diffraction peaks are highly consistent with the standard diffraction patterns of CuS (JCPDS No. 02-0820) and MoS_2_ (JCPDS No. 02-1133), indicating their high purity and excellent crystallinity [32,33,34,35]. Further analysis of MoS_2_/CuS confirmed the coexistence of characteristic peaks for MoS_2_ and CuS. The results fully verify the successful fabrication of the MoS_2_/CuS composite heterostructure. Furthermore, FTIR spectroscopy (Figure 1b) was employed to elucidate the chemical bonding information of the prepared samples. For pure MoS_2_, the broad absorption band located at 400–1000 cm^−1^ originates from the stretching vibration of Mo-S bonds, which corroborates the intrinsic structural features of MoS_2_ [36]. For pure CuS, the intense absorption signal in the range of 1600 cm^−1^ is attributed to the typical vibration of Cu-S bonds, consistent with the standard crystal structure of copper sulfide [37]. As for the MoS_2_/CuS, the characteristic bands of both Mo-S and Cu-S bonds were observed in the 400–1600 cm^−1^ region, demonstrating the successful integration of MoS_2_ and CuS. Importantly, slight peak shifts and broadening of Mo-S and Cu-S characteristic vibrations were detected in the composite compared with pure counterparts. This phenomenon reveals the formation of strong interfacial interactions between MoS_2_ and CuS, rather than simple physical mixing. The above structural and bonding results comprehensively validate the successful construction of the MoS_2_/CuS composite heterostructure.

**Figure 2:**
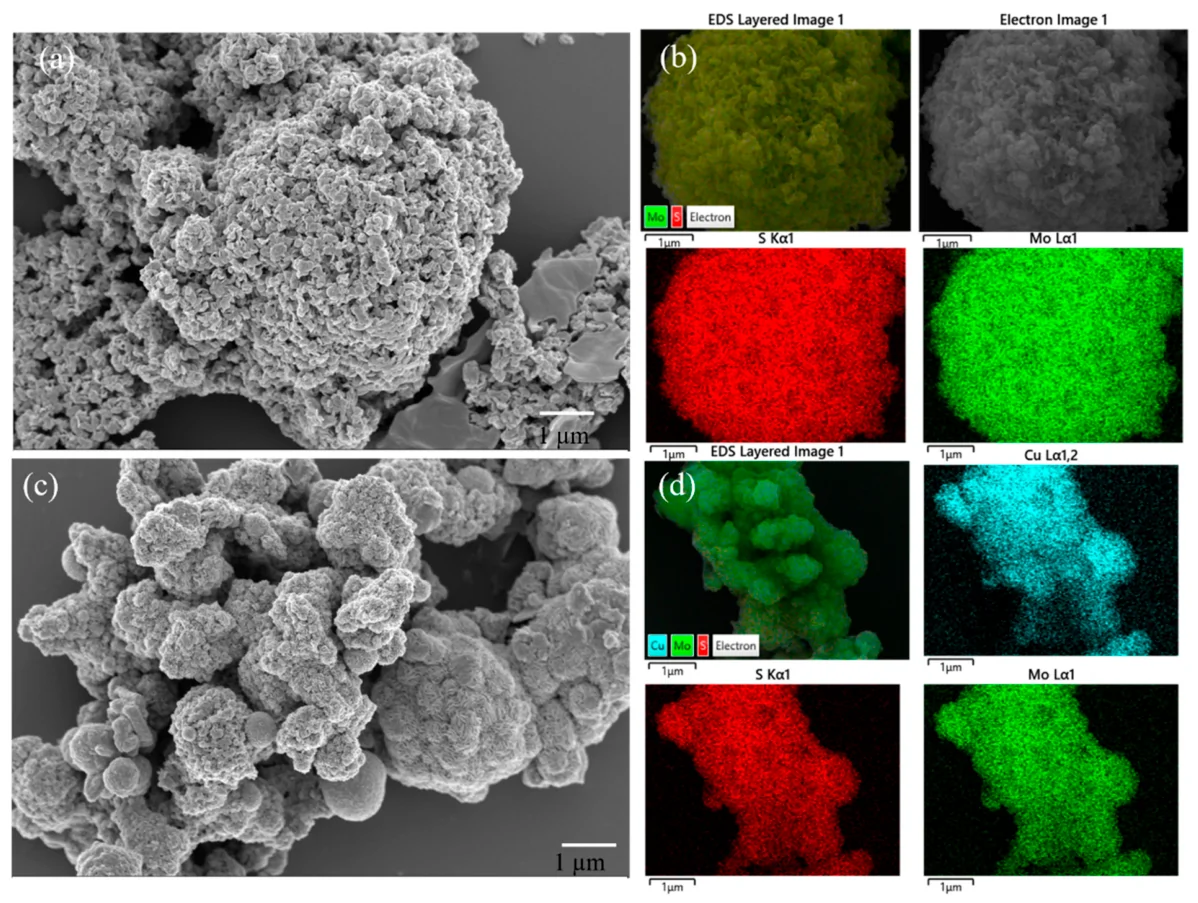
SEM-EDS mapping of MoS_2_ (**a**,**b**) and MoS_2_/CuS (**c**,**d**) catalysts.

Additionally, SEM combined with EDS mapping was performed to investigate the morphology and elemental distribution of as-prepared catalysts. As shown in Figure 2, pure MoS_2_ exhibits dense spherical-like agglomerates closely stacked by numerous nanoparticles. After the incorporation of CuS, the overall morphology of the material shows no obvious change, and it still maintains spherical aggregates stacked by sheet-like structures. The EDS mapping results demonstrate that Mo, S and Cu elements are homogeneously distributed across the MoS_2_/CuS sample without significant element segregation, confirming that CuS is uniformly incorporated into MoS_2_ and MoS_2_/CuS is successfully fabricated. N_2_ adsorption–desorption measurements were further performed to investigate the pore structure properties of MoS_2_ and MoS_2_/CuS. As depicted in Figure S1, both samples exhibit typical Type-IV isotherms with hysteresis loops, verifying the existence of mesoporous structures. The BET specific surface area of pure MoS_2_ is 16.87 m^2^ g^−1^, which decreases to 3.26 m^2^ g^−1^ for MoS_2_/CuS after CuS incorporation, consistent with the reduction in nitrogen adsorption capacity. Such a large difference in specific surface area may also lead to distinct reaction mechanisms.

### 2.2. Enhanced Catalytic Activity over MoS_2_/CuS

**Figure 3:**
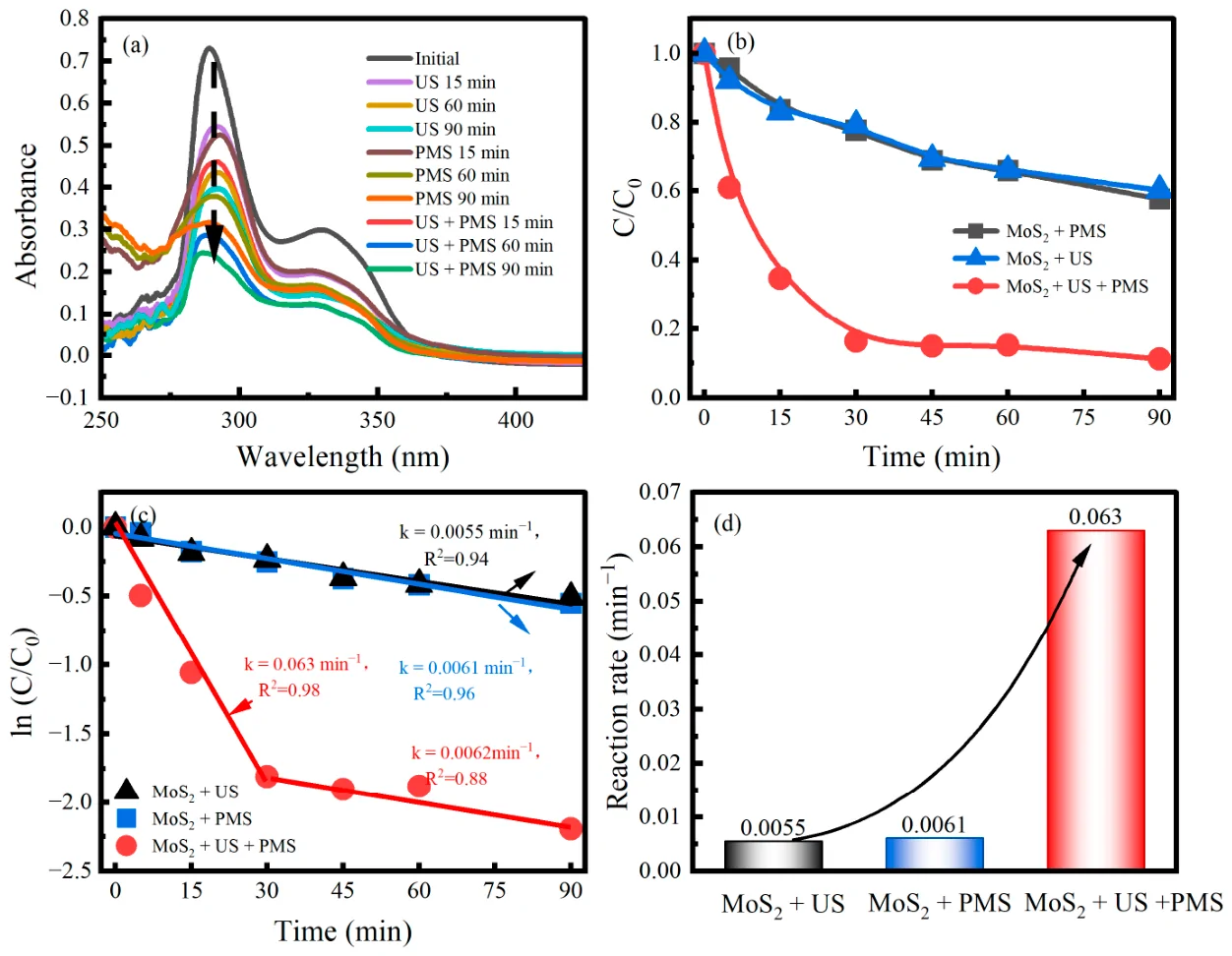
(**a**) UV-Vis absorption spectra under different conditions. (**b**) Variation curves of OFX concentration over time under various reaction conditions. (**c**) Corresponding pseudo-first-order kinetic fitting curves. (**d**) Comparison diagram of reaction rate constants under different conditions.

To explore the regulatory effects of different reaction conditions on the degradation process, MoS_2_ was first used as a catalyst to investigate the degradation performance of OFX under three reaction systems: US alone, PMS alone, and US coupled with PMS (US + PMS). Figure 3a shows the UV-Vis absorption spectra of OFX in different systems after 15 min, 60 min and 90 min of reaction. Compared with the spectrum of the original OFX solution, the intensity of the characteristic absorption peak of OFX in the MoS_2_ + US system remains at a relatively high level. After introducing PMS into the system (MoS_2_ + US + PMS), the concentration of OFX decreases significantly, indicating that PMS and US exhibit a positive synergistic effect during the MoS_2_-catalyzed process, which can effectively promote the degradation of OFX. Figure 3b presents the time-dependent variation curves of OFX degradation efficiency in different systems. It can be observed that the degradation efficiency of OFX is relatively limited in the single systems of MoS_2_ + US and MoS_2_ + PMS. When US and PMS act synergistically in the MoS_2_ + US + PMS system, the catalytic efficiency of MoS_2_ is significantly improved, and the corresponding degradation efficiency increases from approximately 40% in the single systems to about 90%. Further analysis via first-order kinetic fitting (Figure 3c,d) reveals that there is indeed a significant synergistic effect between US and PMS. The degradation rate constant k of the MoS_2_ + US + PMS system is 0.063 min^−1^, which is much higher than those of the MoS_2_ + US system (0.0055 min^−1^) and the MoS_2_ + PMS system (0.0061 min^−1^), and is 5.4 times the sum of the rate constants of the two single systems (0.0116 min^−1^).

**Figure 4:**
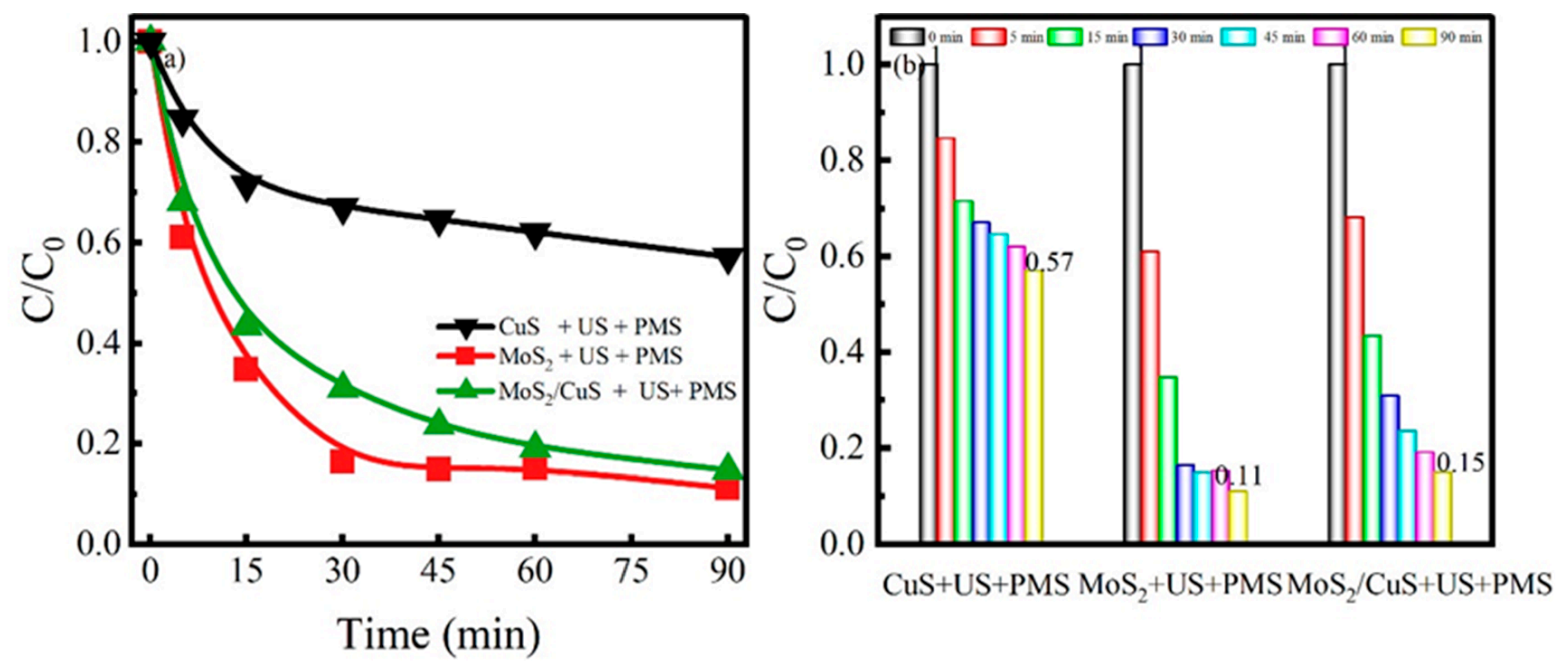
(**a**) Time-dependent curves and (**b**) histogram of OFX degradation efficiency over different materials (CuS, MoS_2_, MoS_2_/CuS) in the ultrasound-assisted peroxymonosulfate system (US + PMS).

Furthermore, the catalytic performance of CuS, MoS_2_ and MoS_2_/CuS composites for PMS activation to degrade OFX with ultrasound assistance was investigated, and the corresponding results are presented in Figure 4. Figure 4a displays the time-dependent curves of OFX degradation efficiency over the three materials in the ultrasound-coupled peroxymonosulfate system. It can be clearly observed that the MoS_2_/CuS composite exhibits remarkably superior OFX removal efficiency compared with pure CuS, and its performance is comparable to that of pristine MoS_2_. Figure 4b is a bar chart comparing the temporal variation in the residual concentration proportion of OFX in different material systems. As illustrated, over 85% of OFX can be eliminated within 90 min in both the pure MoS_2_ and MoS_2_/CuS composite systems, far exceeding the 43% removal efficiency of the CuS system. Although the pure MoS_2_ and MoS_2_/CuS composite systems deliver similar OFX removal efficiencies, their underlying reaction mechanisms may differ. In the MoS_2_ system, adsorption plays a dominant role: OFX is not truly degraded, and the decreased detected concentration merely originates from adsorption. By contrast, reactive species dominate the MoS_2_/CuS composite system, enabling efficient mineralization of OFX rather than simple adsorption.

To further elucidate the degradation behavior of OFX over various catalytic materials (CuS, MoS_2_, MoS_2_/CuS), 3D-EEMs of each reaction system were acquired at different reaction intervals. Two distinct characteristic fluorescence regions were identified from the 3D-EEM spectra of OFX. Zone I exhibited Ex of 200–250 nm and Em of 300–380 nm, deriving from the benzene and quinoline ring fragments of OFX and reflecting the fluorescence signature of its central aromatic skeleton. Zone II ranged from Ex 250–300 nm to Em 350–450 nm, which was assigned to carboxyl, ketone and substituted piperazine functional groups of OFX [38].

**Figure 5:**
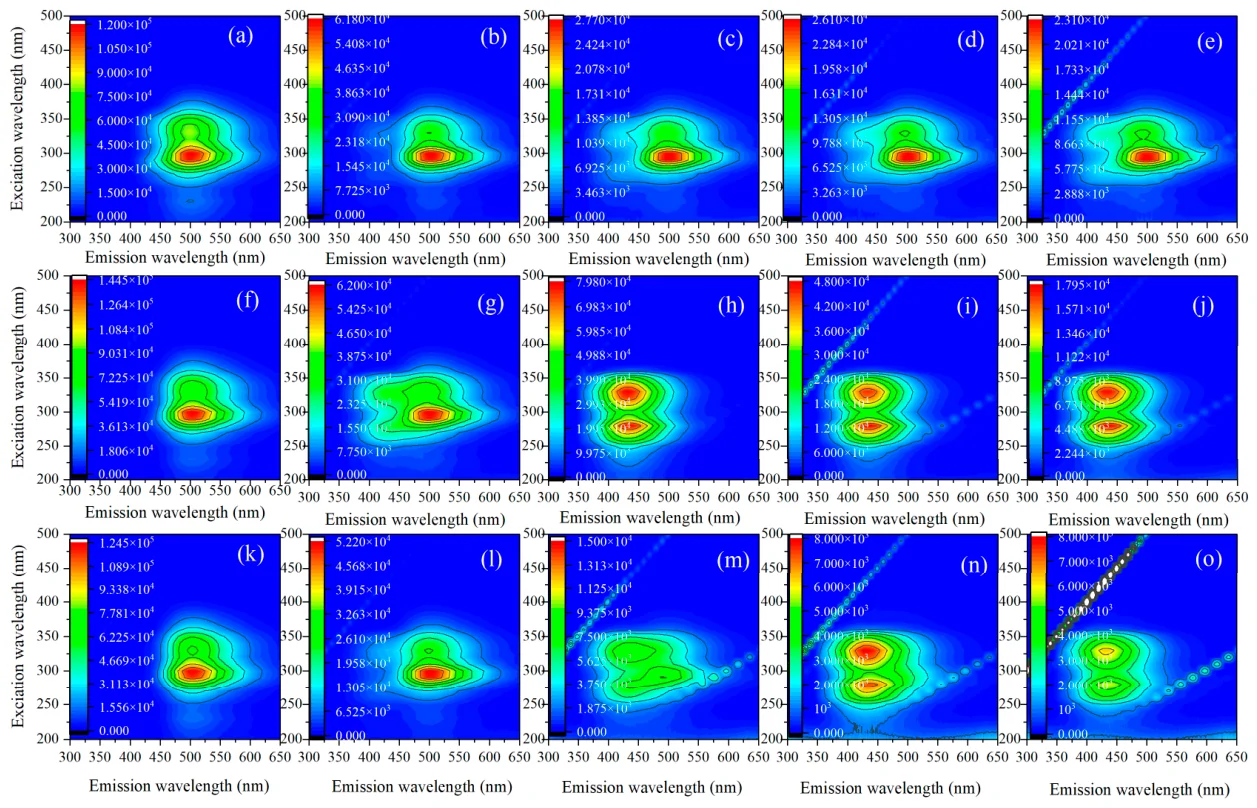
Time-resolved 3D-EEM fluorescence spectra for OFX degradation by different materials (CuS, MoS_2_, MoS_2_/CuS) in ultrasound-coupled peroxymonosulfate system: (**a**–**e**) MoS_2_ at 0, 5, 30, 60 and 90 min; (**f**–**j**) CuS at 0, 5, 30, 60 and 90 min; (**k**–**o**) MoS_2_/CuS composite at 0, 5, 30, 60 and 90 min.

For the MoS_2_ system (Figure 5a–e), the fluorescence coverage area was extensive with intense coloration, which is ascribed to the fact that OFX removal by MoS_2_ is predominantly dependent on physical adsorption or surface complexation. Although this process can rapidly decrease the bulk aqueous concentration of OFX, it fails to effectively cleave the aromatic ring structures of the pollutant. The persistent bright regions (Figure 5a vs. Figure 5e) suggest that OFX is merely sequestered on the MoS_2_ surface, rather than being degraded. For CuS (Figure 5f–j), the overall fluorescence was weaker than that of MoS_2_, but some residual bright areas remained. This can be attributed to the decent catalytic activity of CuS. In the MoS_2_/CuS composite system (Figure 5k–o), white and light-blue regions dominated, while the red cores were both the smallest and rapidly diminished. This clearly shows the higher activity of MoS_2_/CuS, which can be attributed to the synergistic effect of MoS_2_/CuS. MoS_2_ provides a large surface area for OFX adsorption, while CuS provides active sites for PMS activation. The heterojunction interface formed between the two components accelerates charge separation, thereby generating a large quantity of highly oxidizing radicals (^•^OH and SO_4_^•−^) that directly break the benzene rings and conjugated double bonds of OFX. At the end of the reaction (Figure 5o), the contour map was almost entirely converted to blue and green domains, indicating that the molecular structure of OFX had been completely disrupted and transformed into small inorganic molecules or non-fluorescent byproducts, without secondary accumulation of intermediate degradation products. These results not only validate the conclusions derived from the aforementioned degradation experiments, but also further confirm that the MoS_2_/CuS composite exhibits excellent catalytic performance with superior degradation efficiency and stability in the ultrasound-coupled PMS system for the abatement of OFX.

To further verify the above photocatalytic degradation results and assess the deep degradation ability of the catalysts, total organic carbon (TOC) tests were performed. As illustrated in Figure S2, the MoS_2_/CuS catalytic system exhibited a significantly higher TOC removal efficiency than pure MoS_2_ at both 30 min and 90 min of reaction. The gradual improvement in TOC removal efficiency with increasing reaction time demonstrated that the organic intermediates generated during OFX degradation could be continuously decomposed and mineralized. These results strongly confirm that the construction of the MoS_2_/CuS effectively accelerates the deep mineralization of OFX contaminants, thereby achieving superior pollutant removal performance compared with the pristine catalyst.

### 2.3. Optimization of Reaction Parameters

Based on the above results, the MoS_2_/CuS composite exhibits excellent OFX degradation performance under various reaction conditions. Accordingly, the influence of catalyst dosage and PMS dosage on OFX removal efficiency was investigated using OFX as the target pollutant.

**Figure 6:**
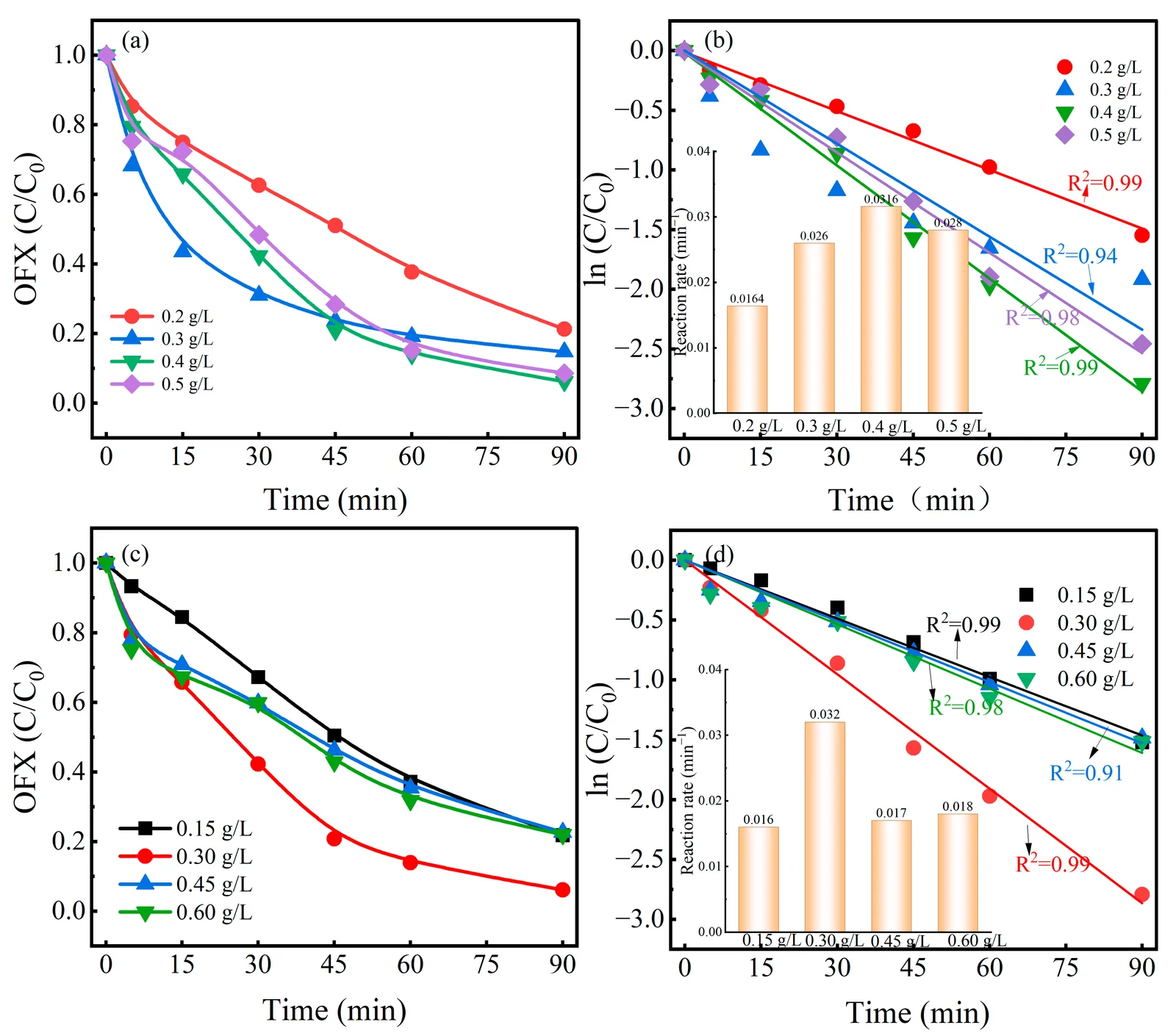
Effects of catalyst dosage on OFX degradation (**a**) and the corresponding degradation kinetic curves (**b**). Effects of PMS concentration on OFX degradation (**c**) and the corresponding degradation kinetic curves (**d**).

From Figure 6a, at a low catalyst dosage of 0.2 g/L, the OFX degradation rate was relatively low, with a pseudo-first-order rate constant of 0.0164 min^−1^. When the dosage increased to 0.3, 0.4, and 0.5 g/L, the 0.4 g/L group achieved the optimal degradation performance, delivering a higher rate constant of 0.0316 min^−1^ than those of other groups. Notably, a further increase in dosage to 0.5 g/L reduced the OFX removal rate instead. Although excessive catalyst dosage provides abundant potential active sites, it inevitably increases the mass transfer resistance of the reaction system. A large number of active sites cannot be fully utilized and remain idle, thereby decreasing the utilization efficiency of reactive species and suppressing OFX degradation.

The MoS_2_/CuS system enables efficient OFX degradation over a wide PMS dosage range, and its catalytic performance is highly PMS dosage-dependent (Figure 6c). At a low PMS dosage of 0.15 g/L, insufficient radical generation resulted in a low OFX removal efficiency of 78% within 90 min and a pseudo-first-order rate constant of only 0.016 min^−1^. Increasing the PMS dosage to 0.30 g/L well matched radical production with pollutant degradation demand, achieving the optimal degradation performance with a maximum rate constant of 0.032 min^−1^ and a 90 min OFX removal efficiency exceeding 94%. Further elevating the PMS dosage to 0.45 g/L and 0.60 g/L adversely suppressed degradation, lowering the removal efficiency to approximately 77% with relatively low-rate constants of 0.017 and 0.018 min^−1^, respectively. The overlapping degradation curves further verify that excessive PMS dosage cannot facilitate OFX degradation. At excessive PMS dosages (0.45–0.60 g/L), surplus HSO_5_^−^ undergoes radical self-quenching with highly reactive SO_4_^•−^ and produces less oxidative SO_5_^•−^, weakening the overall oxidation capacity and reducing both the degradation rate and removal efficiency of OFX. In addition, excess PMS lowers the solution pH, altering the surface charge and active site environment of the catalyst and further restraining catalytic activity. Hence, we selected 0.4 g/L for the MoS_2_/CuS catalyst and 0.30 g/L for PMS as the optimal dosages, considering both degradation efficiency and cost-effectiveness.

### 2.4. Mechanism of OFX Degradation

**Figure 7:**
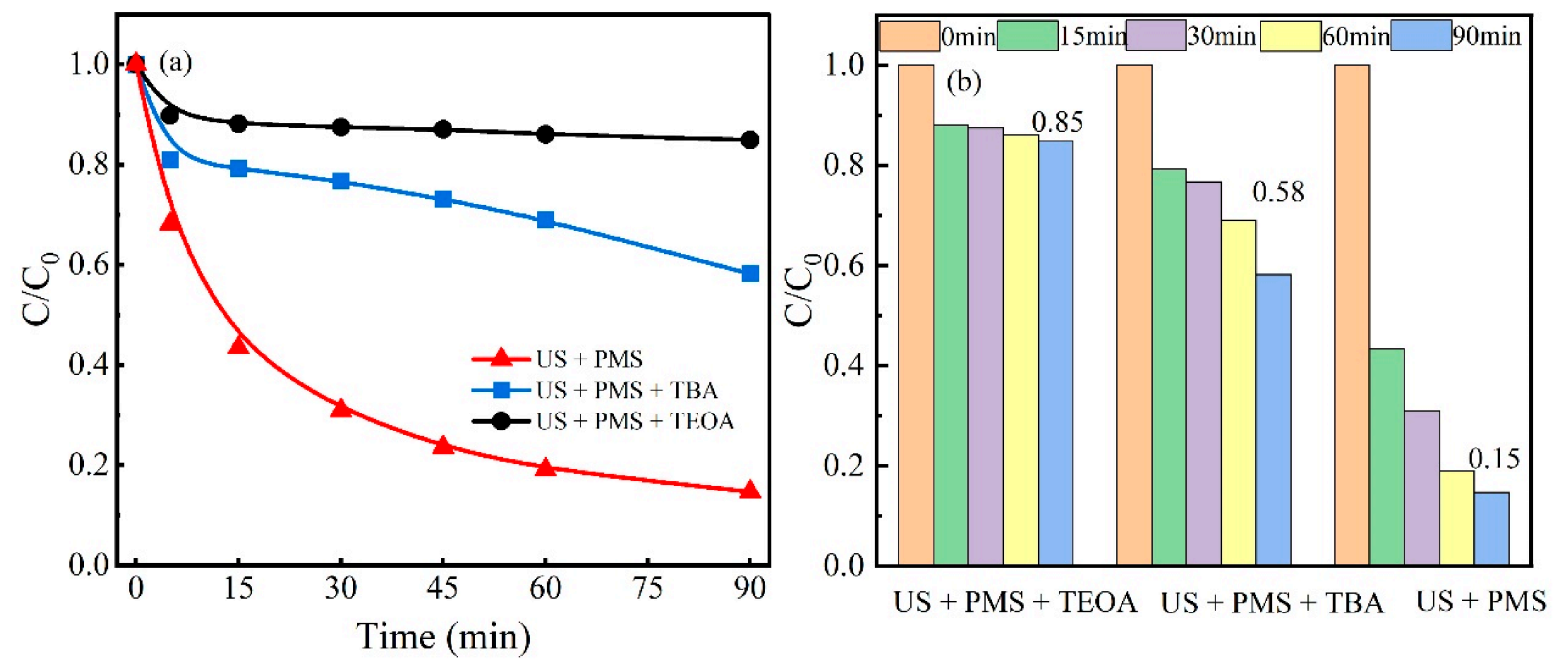
Effects of different active species scavengers on OFX degradation (**a**) and the corresponding histogram of OFX degradation efficiency (**b**).

Previous studies have shown that piezocatalysis produces multiple reactive species, such as ^•^OH. Thus, scavenging experiments using TBA (for ^•^OH/SO_4_^•−^) and TEOA (for h^+^) were conducted to clarify the dominant active species in the MoS_2_/CuS + PMS + US catalytic system [39,40,41,42]. From Figure 7, the removal efficiency of OFX was significantly suppressed after the addition of TBA. The removal efficiency of OFX decreased from 85% to 42% at 90 min, indicating that TBA effectively quenched active radicals in the system, while in the MoS_2_/CuS piezocatalytic system, OFX degradation was completely inhibited after adding TEOA, and its removal efficiency was only 15% within 90 min. The reason lies in TEOA’s efficient scavenging of both ^•^OH and h^+^. The above results confirm that both reactive oxygen radicals and holes are generated in this catalytic system. So, the enhanced catalytic activity can be attributed to two factors. One is the built-in electric field at the MoS_2_/CuS interface, which promotes charge separation, suppresses carrier recombination, and thus boosts PMS activation to generate ^•^OH and SO_4_^•−^ for OFX oxidation. Electrochemical impedance spectroscopy (EIS) was performed to assess the charge-transfer property of the catalysts. As presented in Figure S3, the MoS_2_/CuS heterojunction displays a smaller semicircle radius than pure MoS_2_, implying decreased charge-transfer resistance [43]. Benefiting from the accelerated interfacial charge migration verified by EIS measurement, the synergy between Cu^2+^/Cu^+^ redox couples and intrinsic Mo^4+^/Mo^6+^ sites lowers the energy barrier for O–O bond cleavage in PMS and increases the yield of reactive radicals.

**Figure 8:**
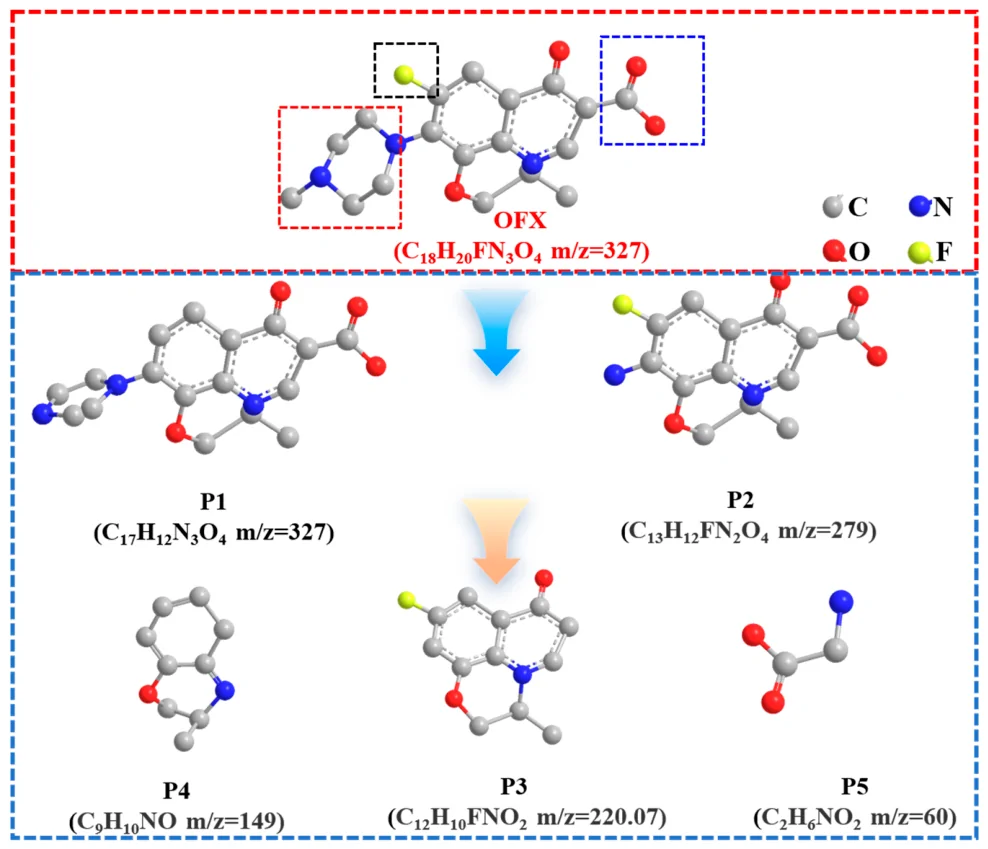
Degradation path analysis of over MoS_2_/CuS catalytic system.

Moreover, the degradation pathway of OFX was measured based on LC-MS. As illustrated in Figure 8 and Figure S4, several major transformation products (P1–P5, corresponding to *m*/*z* = 327, 279, 220, 149 and 60) were successfully detected. And OFX is degraded mainly through three reaction routes: piperazine-ring cleavage, decarboxylation and defluorination. The piperazine moiety is preferentially attacked by reactive species, generating fragmented intermediates. Afterward, decarboxylation and further ring-opening reactions occur, and large aromatic structures are decomposed into small-molecule fragments [44,45,46].

### 2.5. Implications of MoS_2_/CuS Based Fenton Reaction to Water Treatment

**Figure 9:**
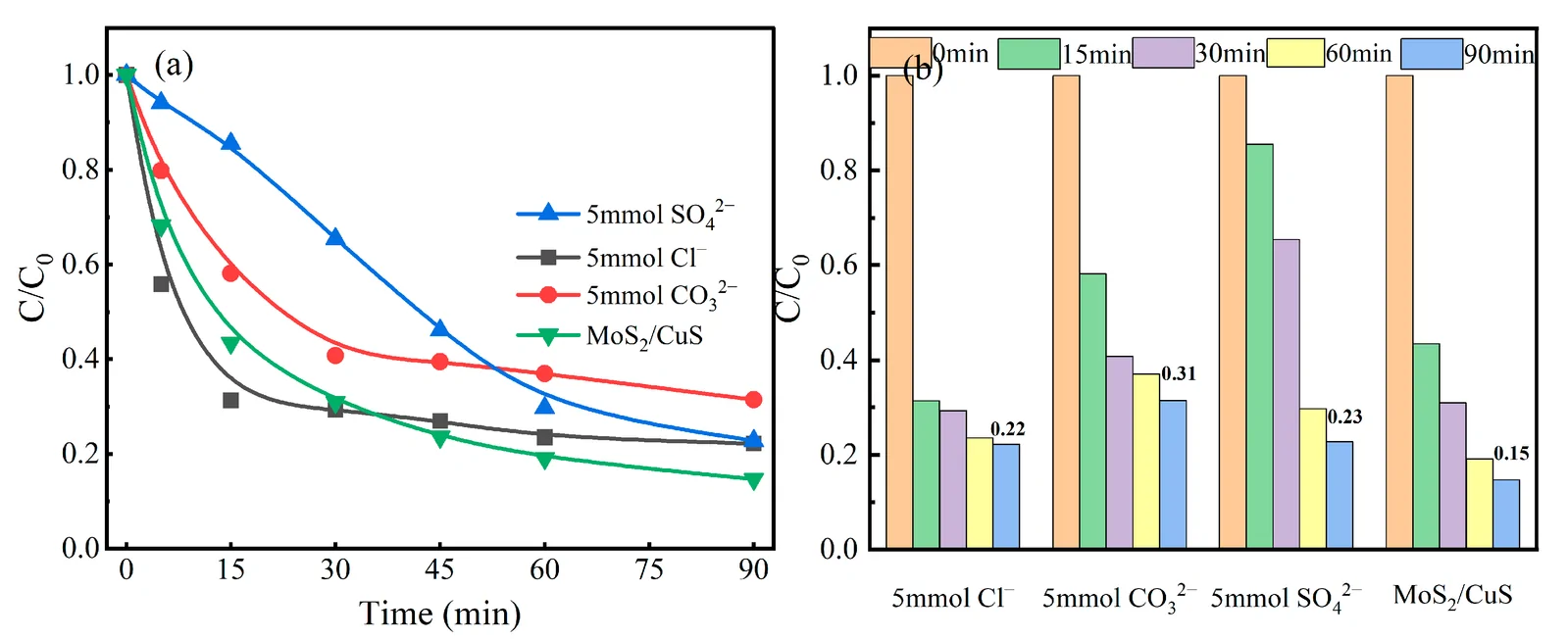
Effects of different anions on OFX degradation (**a**) and histogram of OFX degradation efficiency by Cl^−^, CO_3_^2−^, and SO_4_^2−^ in US + PMS system (**b**). (**a**) shows that coexisting anions suppressed its degradation, with inhibition following the order: CO_3_^2−^ > Cl^−^ > SO_4_^2−^. From (**b**), the removal efficiency of the anion-free blank group reaches 85% at 90 min; the presence of Cl^−^, CO_3_^2−^ and SO_4_^2−^ reduces the removal efficiency to 79%, 68% and 77%, respectively.

To assess the practical application potential of MoS_2_/CuS, the influence of common anions on OFX removal was investigated. Figure 9a shows that coexisting anions suppressed its degradation, with inhibition following the order: CO_3_^2−^ > Cl^−^ > SO_4_^2−^. From Figure 9b, the removal efficiency of the anion-free blank group reaches 85% at 90 min; the presence of Cl^−^, CO_3_^2−^ and SO_4_^2−^ reduces the removal efficiency to 79%, 68% and 77%, respectively. The inhibitory impacts of anions on the MoS_2_/CuS catalytic system stem primarily from radical scavenging, with detailed mechanisms explained below. As potent ^•^OH quenchers, CO_3_^2−^ directly deplete strongly oxidative radicals and drastically lower degradation efficiency, yielding the most severe inhibition. Cl^−^ reacts with ^•^OH to form weakly oxidizing Cl^•^ and Cl_2_^•−^, while competing for catalyst active sites; its inhibitory capacity is inferior to CO_3_^2−^. SO_4_^2−^ barely reacts with ^•^OH and exhibits negligible site competition, leading to the weakest suppression toward OFX degradation [47,48,49]. Overall, although coexisting anions exert certain adverse effects on pollutant elimination, the OFX removal efficiency can still remain at approximately 80%. In addition, recycling tests reveal that the MoS_2_/CuS maintains favorable degradation performance after four cycles (Figure S5), demonstrating its acceptable reusability. Slight activity decay is observed with increasing cycles, which may originate from active-site loss and metal leaching (Figure S6) during repeated reactions [50]. Overall, these results demonstrate that the MoS_2_/CuS possesses acceptable reusability.

Furthermore, the catalytic degradation performance toward various pollutants was investigated using RhB and MB as model contaminants under uniformly optimized conditions to further evaluate the practical application potential of MoS_2_/CuS composites. As illustrated in Figure S7a, bare MoS_2_ exhibited divergent catalytic activity toward the two dyes. RhB was rapidly degraded with a removal efficiency over 95% at 30 min. In contrast, MB degradation proceeded slowly, retaining a removal efficiency of 45% within 90 min. Figure S7b shows that MoS_2_/CuS removed over 80% of both MB and RhB within 90 min, indicating its potential for water treatment.

## 3. Experimental

### 3.1. Chemicals

Chemicals including Cu (NO_3_)_2_·3H_2_O, (NH_4_)_6_Mo_7_O_24_•4H_2_O, thiourea, ethanol, polyvinylpyrrolidone (PVP), peroxymonosulfate (PMS)*,* tert-butyl alcohol (TBA), and triethanolamine (TEOA) were supplied by Sinopharm Chemical Reagent Co., Ltd. (Shanghai, China).

### 3.2. Preparation of MoS_2_/CuS

The detailed synthesis procedure for MoS_2_/CuS composite is described as follows: Firstly, 0.4 g of PVP was dissolved in 50 mL of deionized water under ambient conditions. Then, 5 mmol Cu (NO_3_)_2_·3H_2_O and 0.71 mmol (NH_4_)_6_Mo_7_O_24_·4H_2_O were introduced into the aqueous solution with continuous mechanical stirring. After that, the resulting light-green precipitate was recovered via centrifugation and washed with deionized water and ethanol in sequence. The obtained precipitate was dried at 60 °C for 6 h to yield the copper-molybdenum precursor.

Subsequently, the copper-molybdenum precursor was redispersed in 60 mL of deionized water, after which thiourea was added under continuous stirring for 0.5 h. During this process, the suspension gradually turned yellow. The yellow suspension was transferred into an autoclave and maintained at 200 °C for 24 h. Upon reaction completion, the autoclave was cooled naturally to room temperature. The solid product was separated by centrifugation, repeatedly washed with distilled water and ethanol to eliminate residual impurities, and dried at 60 °C overnight. After gentle grinding, the target MoS_2_/CuS composite powder was finally obtained.

### 3.3. Analytical Methods

#### 3.3.1. Characterization of MoS_2_/CuS

X-ray diffraction (XRD) was employed to analyze the lattice morphology of MoS_2_/CuS over the 2θ range of 10–80° using an X-ray diffractometer (Bruker, Billerica, MA, USA). Fourier transform infrared (FTIR) spectrum was obtained using Fourier transform infrared spectrometer (IR-7600, Tianjin GangDong Co., Ltd., Tianjin, China). Scanning electron microscopy (SEM) combined with elemental mapping was employed to observe the morphology and elemental distribution of the prepared catalysts. The specific surface area and pore structure properties of the catalyst were measured via Brunauer–Emmett–Teller (BET, ASAP2020, Micromeritics, Norcross, GA, USA) nitrogen adsorption–desorption tests. Electrochemical AC impedance spectroscopy tests were carried out to evaluate the interfacial charge transfer efficiency and electrochemical impedance characteristics of the catalyst (Text S1).

#### 3.3.2. Analysis of Degradation Process

Generally, all catalytic degradation experiments were performed at room temperature. First, approximately 0.03 g of the catalyst was dispersed in 100 mL of 10 mg/L OFX solution and sufficiently stirred to establish adsorption equilibrium. Then, 0.3 g/L PMS was added and ultrasonic irradiation at 40 kHz was applied simultaneously to initiate the degradation reaction. Within the given time, samples were taken out and filtered with a 0.22 μm Millipore membrane for further analysis. The residual concentration of OFX was analyzed by UV-Vis spectroscopy at its characteristic absorption peak of 280 nm. In addition, three-dimensional excitation-emission matrix fluorescence spectroscopy (3D-EEMs, HITACHI F-7000, Tokyo, Japan) was employed to investigate the evolution of degradation products. The instrumental parameters were set as follows: the scanning ranges for excitation and emission wavelengths were 200–500 nm and 300–650 nm, respectively. Both excitation and emission slit widths were fixed at 5 nm, and the scanning speed was 12,000 nm/min [38]. Inductively coupled plasma mass spectrometry (ICP-MS, Agilent 7900, Santa Clara, CA, USA) was utilized to quantitatively detect the leaching concentration of copper ions after catalytic reaction, so as to evaluate the structural stability and metal dissolution behavior of the MoS_2_/CuS catalyst. Liquid chromatography-mass spectrometry (LC-MS, Ultimate 3000, Thermo Fisher Scientific, Waltham, MA, USA) was further performed to identify the intermediate products generated during OFX degradation (Text S2). The molecular structures of the degradation byproducts were analyzed accurately, which helped to clarify the possible photocatalytic degradation pathway of the target pollutant.

Furthermore, gradient experiments involving catalyst loading, PMS dosage, coexisting inorganic anions and different pollutants were conducted to explore their influences on OFX degradation efficiency, so as to screen the optimal reaction parameters and offer theoretical guidance for the practical application of the MoS_2_/CuS composite. Finally, to identify the dominant active species in the reaction, the free radical trapping method was employed, where 0.6 M TBA and 10 mM TEOA were added to capture hydroxyl radicals (^•^OH) and holes (h^+^), respectively, and the main active species were determined by comparing OFX degradation efficiency before and after the addition of trapping agents.

## 4. Conclusions

In this work, MoS_2_/CuS material was successfully prepared via a facile hydrothermal method. Compared with pure MoS_2_, the composites exhibited superior piezoelectric catalytic performance for the degradation of OFX. The optimal reaction conditions were determined as the catalyst dosage of 0.4 g/L and PMS dosage of 0.3 g/L. Radical quenching tests verified that •OH and h^+^ were the dominant active species in this catalytic system. The tightly bonded heterojunction interface of MoS_2_/CuS constructs a built-in electric field, which effectively promotes the separation and migration of charge carriers and significantly activates PMS to generate abundant radicals. Meanwhile, the reversible Cu^2+^/Cu^+^ redox cycle induced by CuS can synergistically cooperate with the intrinsic Mo^4+^/Mo^6+^ redox pairs of MoS_2_. This synergistic effect reduces the energy barrier for the cleavage of PMS peroxide bonds, improves the generation efficiency of active radicals, and remarkably enhances its catalytic performance of the MoS_2_/CuS composite. Moreover, the composite catalyst possesses excellent resistance to aqueous ion interference and can achieve efficient and stable degradation of various pollutants, demonstrating great potential for practical environmental remediation applications.

## Data Availability

The original contributions presented in this study are included in the article/Supplementary Material. Further inquiries can be directed to the corresponding authors.

## Supplementary Materials

The following supporting information can be downloaded at: https://www.mdpi.com/article/10.3390/molecules31183210/s1. Text S1. LC MS measurement for ofloxacin. Text S2. Electrochemical AC impedance spectroscopy test method for catalysts. Figure S1. N_2_ adsorption–desorption isotherms and corresponding BJH pore size distribution curves of the as prepared MoS_2_ (a) and MoS_2_/CuS composites (b). Figure S2. The TOC removal efficiency over different catalytic systems. Figure S3. Nyquist plots for MoS_2_ and MoS_2_/CuS. Figure S4. LC-MS spectrum analysis in MoS_2_/CuS catalytic reaction. Figure S5. Evaluation of the cycling performance of MoS_2_/CuS. Figure S6. Leaching amount of copper ions during this catalytic system. Figure S7. Degradation curves of MB and RhB by different catalytic systems: (a) MoS_2_ + US + PMS; (b) MoS_2_/CuS + US + PMS.
