# Supplementary material for: Synergistic Catalysis over MoS2/CuS in Ultrasound-Assisted Peroxymonosulfate System: Performance and Mechanism for Degradation of Multiple Organic Contaminants
Source: Molecules. 2026 Sep 11;31(18):3210. doi: 10.3390/molecules31183210 (PMC13608920; doi:10.3390/molecules31183210)
Supplement: Supplementary file 1 [file molecules-31-03210-s001.zip › molecules-4518491-supplementary.pdf]

Supplementary Materials

Synergistic Catalysis over MoS_2/CuS in Ultrasound-Assisted Peroxymonosulfate System: Performance and Mechanism for Degradation of Multiple Organic Contaminants

Chu Dai [1,2,*], Jie Li [1,2], Chuanhui Wang [1,2], Hongyan Qi [1,2] and Chen Tian [3,*]

[1] College of Advanced Energy Materials, Hubei University of Education, Wuhan 430205, China

[2] Hubei Engineering Technology Research Center of Environmental Purification Materials, Institute of Materials Research and Engineering, Hubei University of Education, Wuhan 430205, China

[3] Yangtze River Fisheries Research Institute, Chinese Academy of Fishery Sciences, Wuhan 430223, China

* Correspondence: chudai528@126.com (C.D.); tianchen@yfi.ac.cn (C.T.)

There are 8 pages: 2 Texts and 7 Figures.

Text S1. LC-MS measurement for ofloxacin

Text S2. Electrochemical AC impedance spectroscopy test method for catalysts

Figure S1. N_2 adsorption–desorption isotherms and corresponding BJH pore-size distribution curves of the as-prepared MoS_2 (a) and MoS_2/CuS composites (b).

Figure S2. The TOC removal efficiency over different catalytic systems.

Figure S3. Nyquist plots for MoS_2 and MoS_2/CuS.

Figure S4. LC-MS spectrum analysis in MoS_2/CuS catalytic reaction.

Figure S5. Evaluation of the cycling performance of MoS_2/CuS.

Figure S6. Leaching amount of copper ions during this catalytic system.

Figure S7. Degradation curves of MB and RhB by different catalytic systems: (a) MoS_2 + US + PMS; (b) MoS_2/CuS + US + PMS.

Text S1. LC-MS measurement for ofloxacin

Ofloxacin was quantified by LC-MS (Ultimate 3000 UHPLC -Q Exactive). Chromatographic separation was performed on an ACQUITY UPLC BEH C18 column (1.7 μm, 2.1 × 50 mm). Mobile phases consisted of water with 0.1 % formic acid (A) and acetonitrile with 0.1 % formic acid (B). The flow rate was 0.4 mL/min, injection volume was 5 μL, and column temperature was kept at 40 °C. Gradient elution: 0 min, 5 % B; 2.5 min, 95 % B; 3.0 min, 95 % B; 3.1 min, 5 % B. Mass spectrometry was operated in ESI positive-ion mode. Capillary voltage: 3.5 kV; source temperature: 150 °C; desolvation temperature: 400 °C; cone gas flow: 50 L/h; desolvation gas flow: 800 L/h. Multiple reaction monitoring (MRM) was used for data acquisition.

Text S2. Electrochemical AC impedance spectroscopy test method for catalysts

For the electrochemical test of catalyst A, 10 mg of catalyst A powder was dispersed in a mixed solution of 950 μL water and ethanol, and 50 μL Nafion solution was subsequently added to the dispersion. The mixture was ultrasonically treated for 15 min to form a uniformly dispersed catalyst suspension. Afterward, 10 μL of the homogeneous suspension was drop-cast on the surface of pre-cleaned glassy carbon electrodes (GCE) and dried naturally at room temperature for subsequent electrochemical tests. All electrochemical tests of catalysts were carried out on a CHI760E electrochemical workstation (Chenhua Instrument, Shanghai, China) equipped with a standard three-electrode system. A 0.1 M $NasO_4$ aqueous solution was used as the electrolyte. A platinum electrode was applied as the counter electrode, and an Ag/AgCl electrode was used as the reference electrode throughout the measurements.

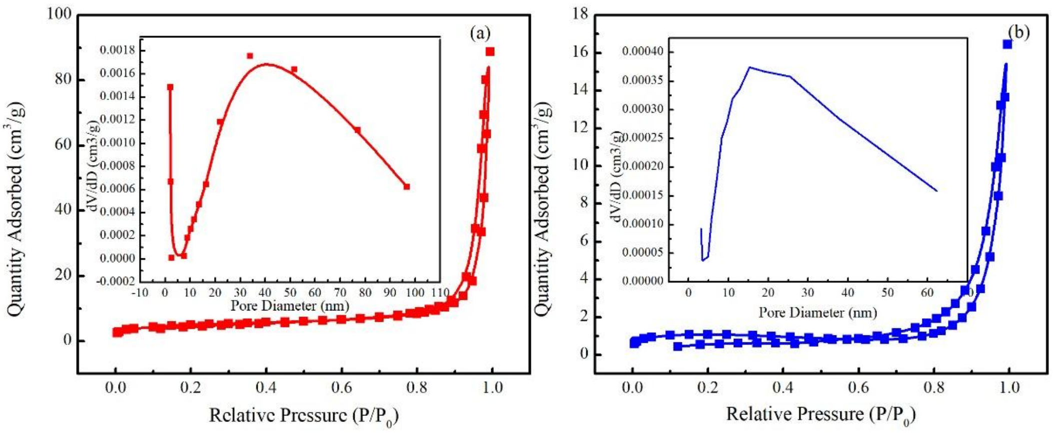

Figure S1. N_2 adsorption–desorption isotherms and corresponding BJH pore-size distribution curves of the as-prepared MoS_2 (a) and MoS_2/CuS composites (b).

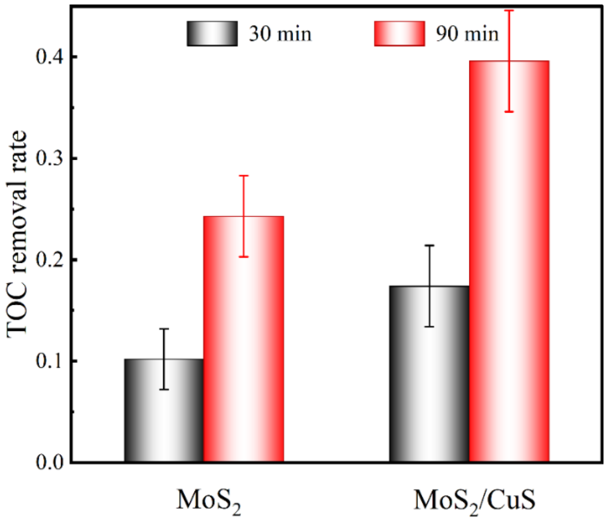

Figure S2. The TOC removal efficiency over different catalytic systems.

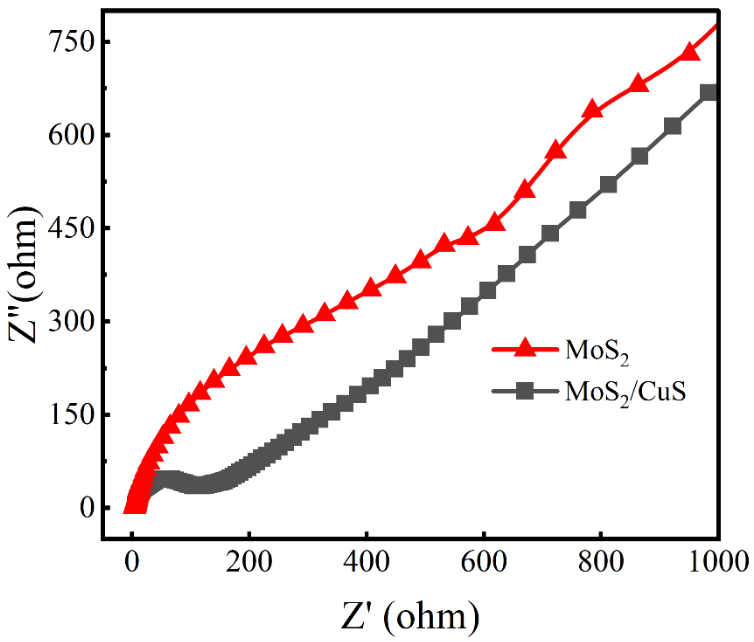

Figure S3. Nyquist plots for MoS_2 and MoS_2/CuS.

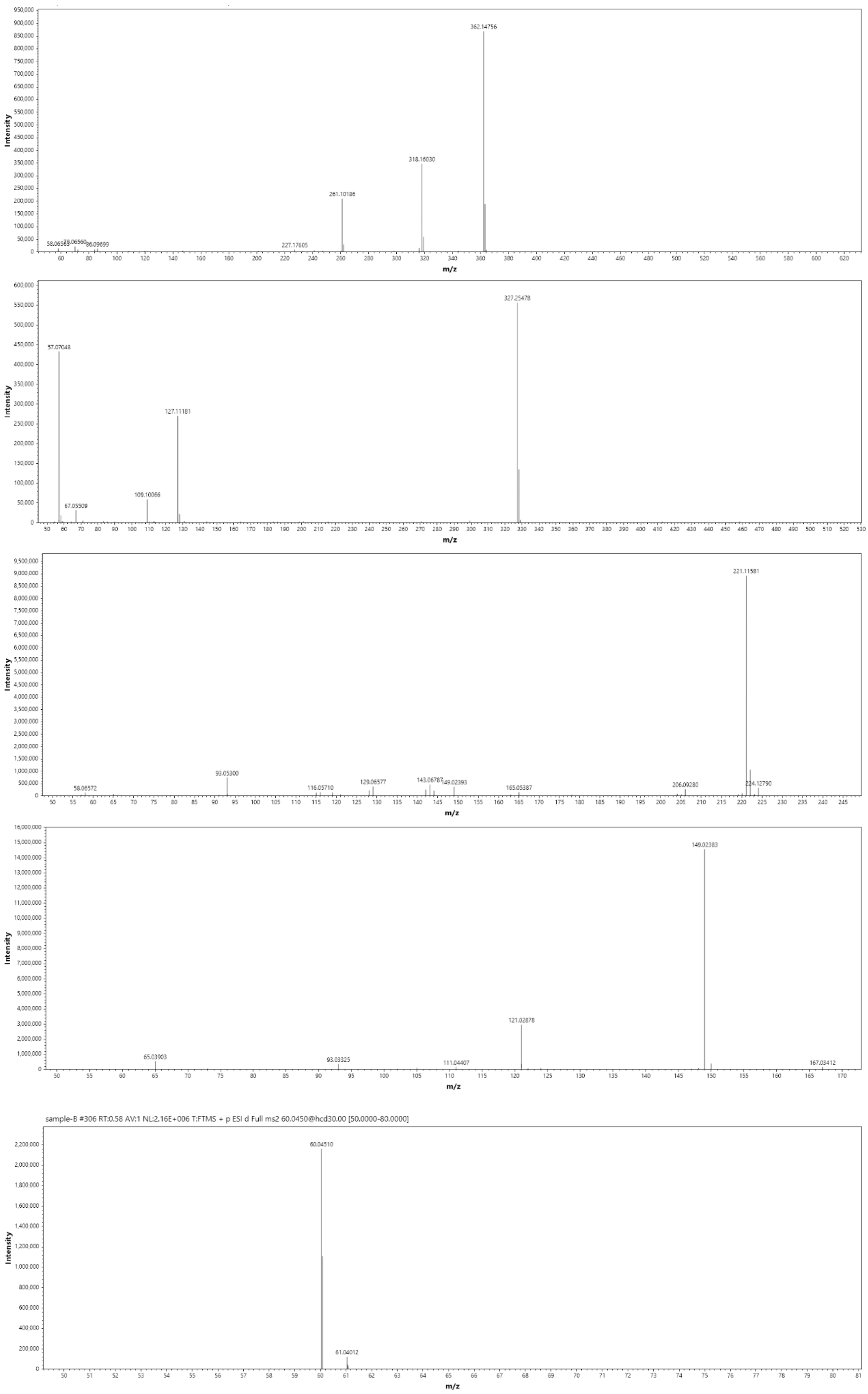

Figure S4. LC-MS spectrum analysis in MoS_2/CuS catalytic reaction.

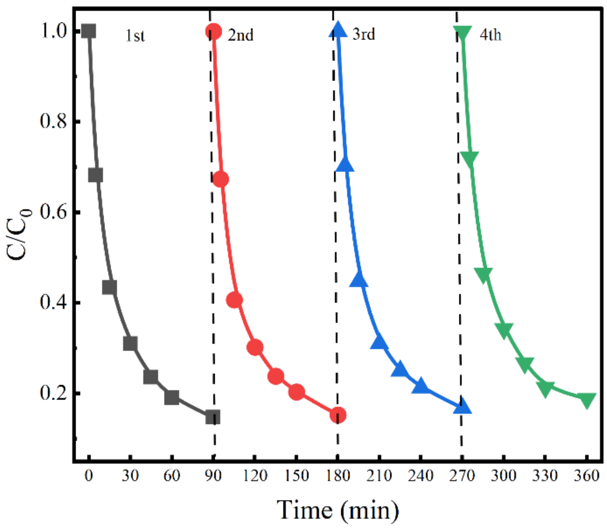

Figure S5. Evaluation of the cycling performance of MoS_2/CuS.

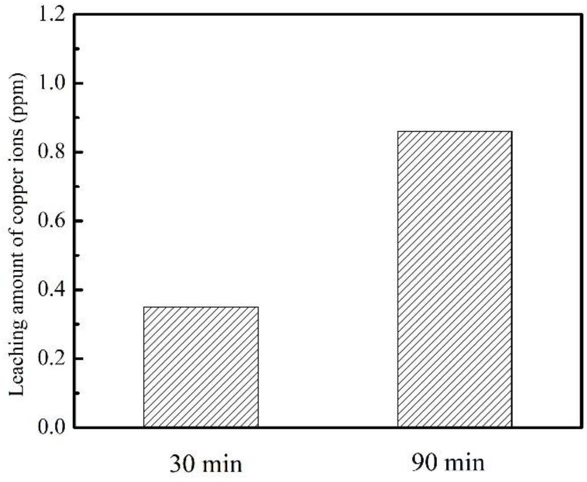

Figure S6. Leaching amount of copper ions in this catalytic system.

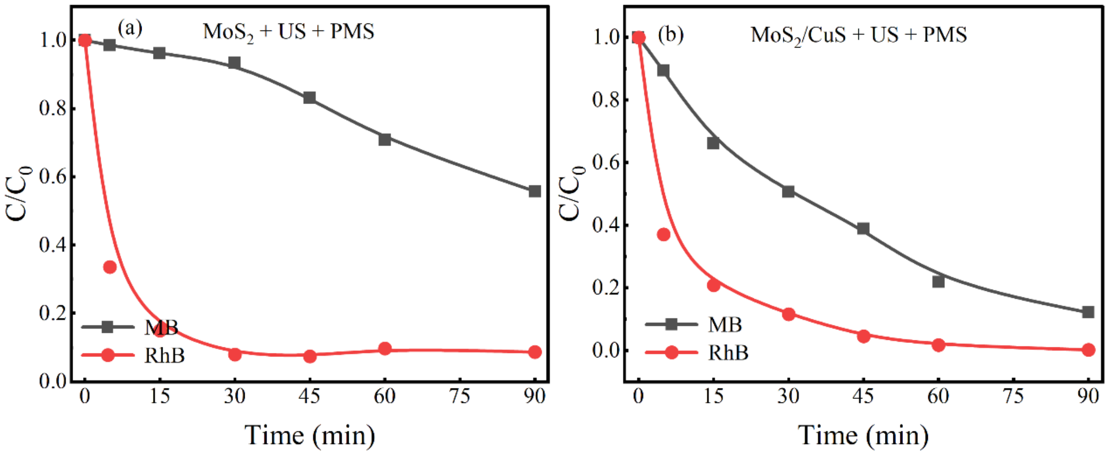

Figure S7. Degradation curves of MB and RhB by different catalytic systems: (a) MoS_2 + US + PMS; (b) MoS_2/CuS + US + PMS.
